# [Corrigendum] Knockdown of lncRNA C5orf66‑AS1 inhibits osteosarcoma cell proliferation and invasion via miR‑149‑5p upregulation

**DOI:** 10.3892/ol.2025.15000

**Published:** 2025-03-31

**Authors:** Hui Zhang, Jie Song

Oncol Lett 22: 757, 2021; DOI: 10.3892/ol.2021.13018

Following the publication of the above article, the authors contacted the Editorial Office to explain that they had made some errors in labelling the different experimental groups associated with the flow cytometric plots shown in Fig. 5B on p. 5, which had resulted in the data in this figure being assembled incorrectly in the published paper. The authors requested that they might publish a corrected version of Fig. 5, showing data from one of their repeated experiments for Fig. 5B, in a Corrigendum.

Fig. 5, which has been revised along these lines, is shown below. The authors regret that these errors occurred. All the authors agree with the publication of this Corrigendum, and thank the Editor of *Oncology Letters* for granting them the opportunity to publish this; furthermore. they apologize to the readership for any inconvenience caused.

## Figures and Tables

**Figure 5.**
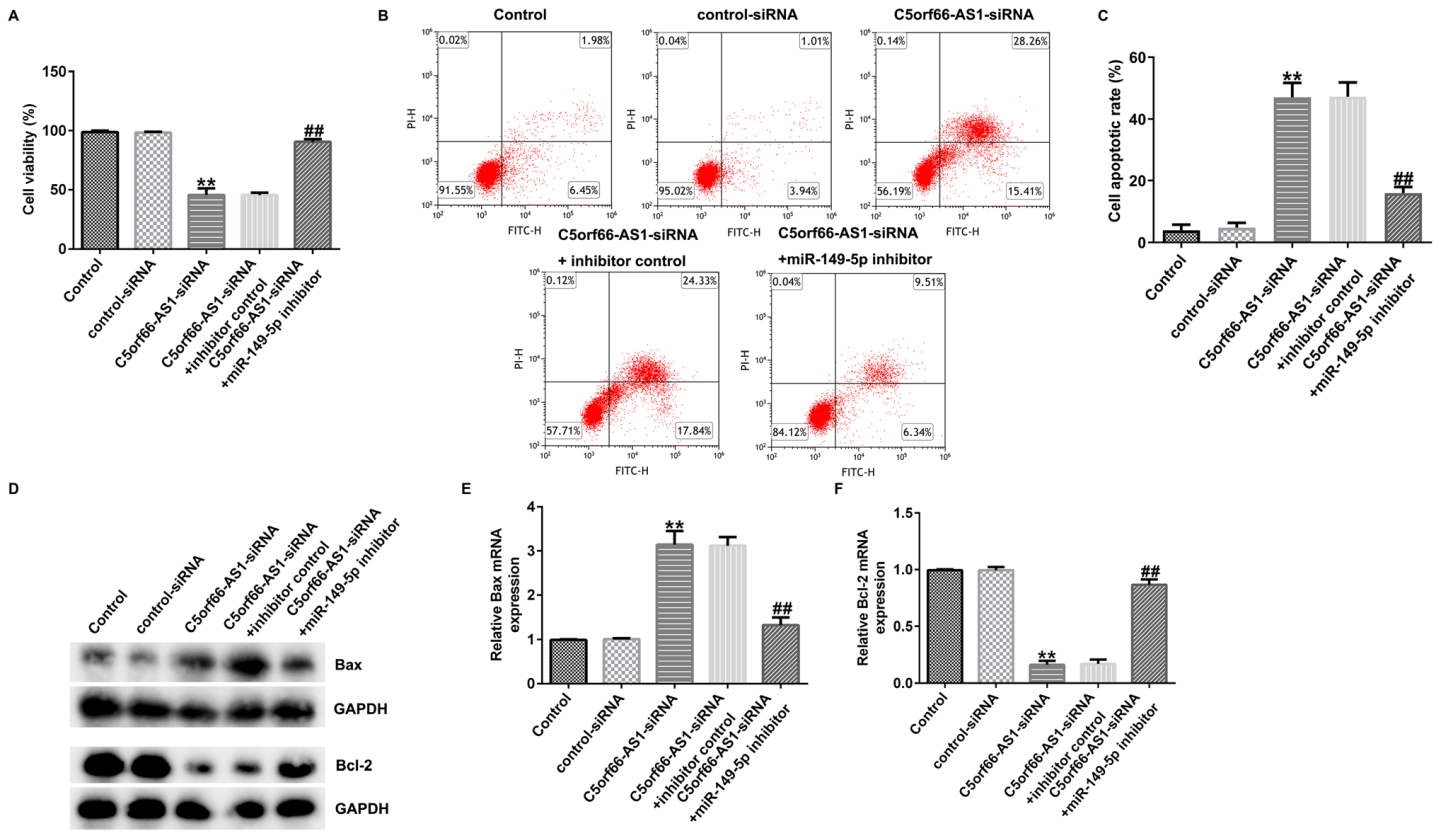
Effects of C5orf66-AS1-siRNA on OS cell proliferation and apoptosis. U2OS cells were transfected with control-siRNA, C5orf66-AS1-siRNA, C5orf66-AS1-siRNA+inhibitor control or C5orf66-AS1-siRNA+miR-149-5p inhibitor for 48 h. (A) Cell Counting Kit-8 assay was used to assess cell viability. (B) Apoptotic rate of U2OS cells was evaluated by flow cytometry. (C) Quantification of the apoptotic cell rate. (D) Western blot analysis of Bax or Bcl-2 protein expression. Expression levels of (E) Bax and (F) Bcl-2 in U2OS cells were determined by RT-qPCR. **P<0.01 vs. control-siRNA; ^##^P<0.01 vs. C5orf66-AS1-siRNA+inhibitor control. miR, microRNA; OS, osteosarcoma; si, small interfering; RT-qPCR, reverse transcription-quantitative PCR, CCK-8, Cell Counting Kit-8; C5orf66-AS1, C5orf66-antisense 1.

